# Cathepsin F and Fibulin-1 as novel diagnostic biomarkers for brain metastasis of non-small cell lung cancer

**DOI:** 10.1038/s41416-022-01744-3

**Published:** 2022-02-25

**Authors:** Song Wei, Wenwen Liu, Mingxin Xu, Huamin Qin, Chang Liu, Rui Zhang, Sihai Zhou, Encheng Li, Zhiyu Liu, Qi Wang

**Affiliations:** 1 Department of Respiratory Medicine, The Second Hospital, Dalian Medical University, Dalian, China; 2 Department of Oncology, Beijing Chest Hospital, Capital Medical University, Beijing, China; 3 Cancer Translational Medicine Research Center, The Second Hospital, Dalian Medical University, Dalian, China; 4 Department of Pathology, The Second Hospital, Dalian Medical University, Dalian, China; 5 Department of Urology Surgery, The Second Hospital, Dalian Medical University, Dalian, China

**Keywords:** Non-small-cell lung cancer, Predictive markers, Non-small-cell lung cancer, Diagnostic markers

## Abstract

**Background:**

The lack of non-invasive methods for detection of early micro-metastasis is a major cause of the poor prognosis of non-small cell lung cancer (NSCLC) brain metastasis (BM) patients. Herein, we aimed to identify circulating biomarkers based on proteomics for the early diagnosis and monitoring of patients with NSCLC BM.

**Methods:**

Upregulated proteins were detected by secretory proteomics in the animal-derived high brain metastatic lung cancer cell line. A well-designed study composed of three independent cohorts was then performed to verify these blood-based protein biomarkers: the serum discovery and verification cohorts (*n* = 80; *n* = 459), and the tissue verification cohort (*n* = 76). Logistic regression was used to develop a diagnostic biomarker panel. Model validation cohort (*n* = 160) was used to verify the stability of the constructed predictive model. Changes in serum Cathepsin F (CTSF) levels of patients were tracked to monitor the treatment response. Progression-free survival (PFS) and overall survival (OS) were analysed to assess their prognostic relevance.

**Results:**

CTSF and Fibulin-1 (FBLN1) levels were specifically upregulated in sera and tissues of patients with NSCLC BM compared with NSCLC without BM and primary brain tumour. The combined diagnostic performance of CTSF and FBLN1 was superior to their individual ones. CTSF serum changes were found to reflect the therapeutic response of patients with NSCLC BM and the trends of progression were detected earlier than the magnetic resonance imaging changes. Elevated expression of CTSF in NSCLC BM tissues was associated with poor PFS, and was found to be an independent prognostic factor.

**Conclusions:**

We report a novel blood-based biomarker panel for early diagnosis, monitoring of therapeutic response, and prognostic evaluation of patients with NSCLC BM.

## Introduction

Lung cancer is one of the most common causes of cancer-related deaths worldwide [1]. It shows a high propensity for brain metastasis (BM). Non-small cell lung cancer (NSCLC) accounts for 85% of all cases of lung cancer and 30–50% of patients with NSCLC develop BM [1–3]. Patients with BM have an extremely poor prognosis (median survival time: 4–6 months). The lack of methods for detection of early micro-metastasis is a major cause of poor prognosis [4].

Currently, the diagnosis of BM is mainly based on medical imaging and histopathological analysis of samples obtained by biopsy or surgical resection. Routine contrast-enhanced brain magnetic resonance imaging (MRI) for detection of BM is recommended for patients with stage II–IV NSCLC; however, the high cost and low predictive ability for long-term recurrence is a major limitation of MRI [5, 6]. Moreover, MRI scans are dependent on the impairment of the blood-brain barrier, which does not occur until the progression of the disease to a relatively late stage [7]; this limits the application of MRI for early diagnosis. Histopathological analysis of a biopsy or surgical specimens is the gold standard for diagnosis of BM; however, it is a highly invasive procedure and provides limited information about the status of the tumour. Thus, the development of a more sensitive, specific and non-invasive diagnostic approach for BM is a key imperative [8, 9].

Recently, molecular biomarkers of liquid biopsy have been commonly used for screening tumours at an early stage due to its minimally invasive nature, low cost, wider access and good reproducibility. The use of molecular biomarkers to monitor disease progression and therapeutic response can help improve the survival rate of patients with malignant tumours [10–12]. For example, carcinoembryonic antigen (CEA), squamous cell carcinoma antigen (SCC-Ag), carbohydrate antigen 125 (CA125) and cytokeratin fragment 21–1 (CYFRA21-1) have been widely used as serum biomarkers for early diagnosis and assessment of NSCLC [13]. However, there are no specific blood biomarkers of BM originating from lung cancer. Studies have demonstrated fundamental changes in the expression profiles of metastatic tumours and tumours in situ [14]. Therefore, exploring the protein expression profile changes of BM may help identify novel serum biomarkers for the early diagnosis of BM.

Proteomics is a powerful and promising complementary technology that can provide insights into the pathologic changes associated with diseases by screening globular protein alterations to a very sensitive degree [15–17]. In particular, it can help explain the mechanisms by which cellular networks contribute to cancer progression. This approach has been widely used to identify novel biomarkers of various malignant tumours [13, 18, 19]. The approach to secretory proteomics offers promising prospects for screening potential serum biomarkers of diseases. However, this powerful technology is yet to be fully leveraged in the context of lung cancer BM.

In this study, we used quantitative proteomics to screen the potential candidate proteins in a high brain metastasis cell line and verified the expression levels of candidates in the clinical serum and tissue specimens. Cathepsin F (CTSF) and Fibulin-1 (FBLN1), which possess catalytic and binding activities, were identified as novel diagnostic biomarkers for NSCLC BM. We also sought to establish a prediction model for early detection of BM in patients with lung cancer based on the CTSF and FBLN1. We further explored the value of CTSF as a biomarker for therapeutic monitoring and prognostic assessment of patients with lung cancer BM.

## Materials and methods

### Study design and participants

**Fig. 1:**
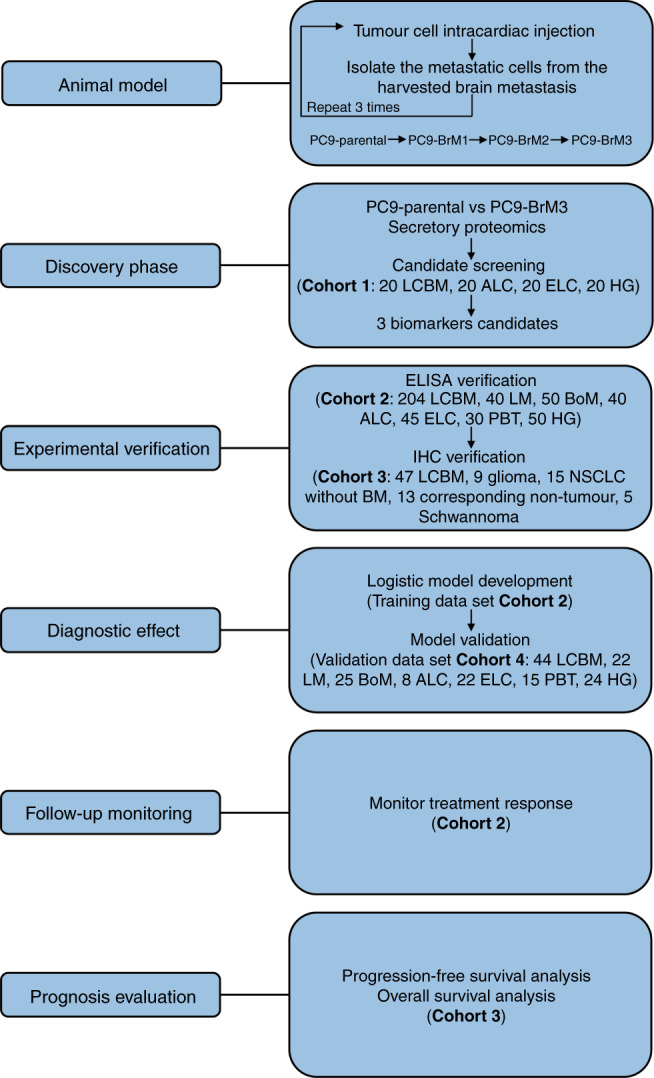
Schematic illustration of the study design. LCBM non-small cell lung cancer (NSCLC) brain metastasis (BM), LM single organ liver metastasis, BoM single organ bone metastasis, ALC advanced NSCLC without distant organ metastasis, ELC early-stage NSCLC, PBT primary brain tumours, HG healthy group. Corresponding non-tumour is matched with patient of NSCLC without BM.

This study consisted of six phases: animal model construction, the discovery phase, experimental verification, assessment of diagnostic performance, follow-up monitoring and prognostic evaluation (Fig. 1 and Supplementary materials and Methods). Participants enrolled in this case-control study were from the Second Affiliated Hospital of the Dalian Medical University, Dalian, China, from 10 January 2016 and 31 October 2020. The diagnosis of NSCLC and primary brain tumour was confirmed by pathology (surgical resection and/or biopsy). A healthy group comprised of outpatients without cancer who underwent a physical examination. All patients with advanced NSCLC and primary brain tumours completed baseline brain MRI examinations at the time of initial diagnosis and prior to receiving anti-tumour therapy. Peripheral blood samples for protein testing were also collected before undergoing anti-tumour therapy. Response Evaluation Criteria in Solid Tumors (RECIST) 1.1 was used to evaluate therapeutic efficacy. Written informed consent was obtained from all subjects prior to their enrolment.

### Animal studies and cell lines culture

Animal models of lung cancer BM were established as previously described [20]; complete details are provided in the supplementary materials and methods. After anesthetising with ketamine (100 mg/kg body weight; Sigma, USA) and xylazine (10 mg/kg body weight; Sigma, USA), brain metastatic subpopulations (PC9-BrM1, PC9-BrM2, and PC9-BrM3) were created by injecting tumour cells PC9 into the left-ventricle of immunodeficient mice and isolating the metastatic cells from the harvested brain metastases. The high brain metastatic lung cancer cell line PC9-BrM3 was established by repeated injection-isolation-expansion cycling three additional times, and cultured in Roswell Park Memorial Institute medium-1640 (RPMI1640) supplemented with 10% FBS, 100 U/mL penicillin and 100 U/mL streptomycin at 37 °C in a humidified atmosphere with 5% CO_2_ (all these reagents were from Gibco, Invitrogen Inc., Carlsbad, California, USA). The human lung cancer cell line PC9 was purchased from the Chinese Academy of Medical Sciences (Beijing, China). Cell lines were regularly authenticated by STR profiling and tested for mycoplasma contamination. Mice were sacrificed by spinal dislocation after the appearance of brain metastases.

### Quantitative tandem mass tag (TMT)-based proteomics

This work was supported by Jingjie PTM BioLab (Hangzhou, China) Co. Ltd. The main experimental procedures of TMT proteomics analysis, including protein extraction, trypsin digestion, TMT labeling, HPLC fractionation, LC-MS/MS analysis and database search are described in the Methods of Supplementary materials in detail.

### Enzyme-linked immunosorbent assay (ELISA)

Serum samples were collected according to standard operating procedures (Supplementary materials). Serum levels were determined using the human CTSF, FBLN1, Aldo-keto reductase family 1 member B10 (AKR1B10) quantitative ELISA kit from Omin.mAbs (Alhambra, California, USA, OM500006, OM527064, OM502039), human C-C motif chemokine 20 (CCL20), Serum amyloid A-1 (SAA1), Growth-regulated alpha (CXCL1), C-X-C motif chemokine 3 (CXCL3) quantitative ELISA kit from Elabscience (Wuhan, China, E-EL-H0027C, E-EL-H2183C, E-EL-H0045C, E-EL-H6008), human Tyrosine-protein kinase receptor UFO (AXL) quantitative ELISA kit from Cusabio (Wuhan, China, CSB-EL002476HU) and human Aldo-keto reductase family 1 member C3 (AKR1C3), and Copine-3 (CPNE 3) quantitative ELISA kit from J&L Biological (Shanghai, China, JL47378-96T, JL50889-96T), according to the manufacturer’s instructions (See Supplementary materials for complete details).

### Immunohistochemistry (IHC)

The expressions of CTSF, FBLN1 and AKR1B10 in tissue samples were examined by IHC. Briefly, tissue sections (3 µm) were deparaffinised, rehydrated, incubated with 3% H2O2 in methanol and subjected to antigen retrieval by EDTA buffer. The sections were blocked with 5% goat serum, probed overnight with primary antibodies for CTSF (1:300, R&D systems, AF2075-SP, Minneapolis, Minnesota, USA), FBLN1 (1:100, Santa Cruz Biotechnology, sc-55470, Heidelberg, Germany), and AKR1B10 (1:500, Abcam, ab192865, Cambridge, UK) at 4 °C. Tissue sections were reacted with biotinylated secondary antibodies and detected by the Streptavidin-Peroxidase IHC assay kit and diaminobenzidine. In each IHC experiment, tissues expressing different antigen levels were included to control the variation between experiments. Two independent pathologists evaluated the immunostaining in a blinded fashion and performed the scoring. They assessed the intensity of staining and the percentage of stained cells (negative staining: 0 points; weak positive staining: 1 point; positive staining: 2 points; strong positive staining: 3 points). We referred to lung cancer on the website (http://www.proteinatlas.org) to establish a positive control for CTSF, FBLN1, and AKR1B10 expression.

### Statistical analysis

All statistical analyses were performed using statistical analysis software SPSS 23.0 at a nominal significance level of 0.05 (two-sided). Between-group differences with respect to serum CTSF, FBLN1, and AKR1B10 levels were assessed using Analysis of variance (ANOVA). The correlation of CTSF, FBLN1 and AKR1B10 expressions in serum or tissues with clinical variables was assessed using *t*-test, ANOVA, Pearson ‘s Chi-squared test, or Fisher exact test. A combined predictive model was developed using logistic regression analysis. The sensitivity and specificity of CTSF and FBLN1 and the optimal cut-off levels for predicting BM in NSCLC patients were determined using receiver operating characteristic curve (ROC) analysis. Progression-free survival (PFS) and overall survival (OS) were estimated by Kaplan–Meier analysis. The log-rank test was used to assess the difference in survival curves between the low and high CTSF expression groups. Cox regression model, hazards ratio (HR) and 95% confidence intervals (CI) were used to evaluate the association between CTSF and the risk of progression in patients with BM. The proportional-hazards assumption was assessed through Time-Dependent Cox Regression Model.

## Results

### Proteomics identified potential diagnostic candidates for NSCLC BM

**Fig. 2:**
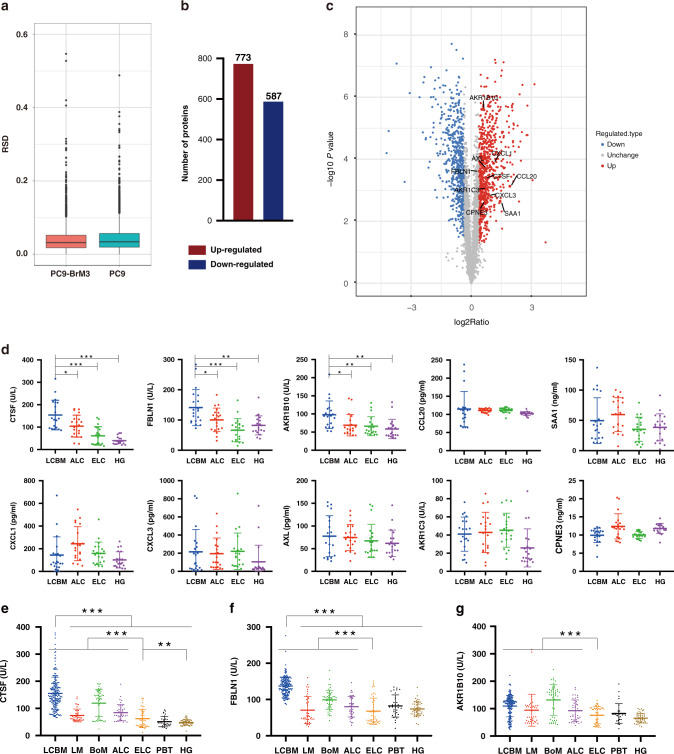
Proteomics was used to screen candidate proteins. **a** The RSD distribution of QC samples. **b** Protein expression profile of PC9-BrM3 compared to parent PC9. **c** Volcano plot shows the upregulation of candidate proteins. **d** Results of enzyme-linked immunosorbent assay (ELISA) showing serum levels of ten candidate proteins in each clinical group in cohort 1. Results of ELISA showing serum levels of Cathepsin F (CTSF) (**e**), Fibulin-1 (FBLN1) (**f**) and Aldo-keto reductase family 1 member B10 (AKR1B10) (**g**) in each clinical group in cohort 2. Each dot corresponds to a single individual. Data are presented as mean ± SD; ***P < 0.001; **P < 0.01; *P < 0.05. LCBM non-small cell lung cancer (NSCLC) brain metastasis (BM), LM single organ liver metastasis, BoM single organ bone metastasis, ALC advanced NSCLC without distant organ metastasis, ELC early-stage NSCLC, PBT primary brain tumours, HG healthy group. The graphs show summarised results from three independent experiments.

In order to explore secretory proteins that are highly expressed during the process of BM of NSCLC, we collected the supernatant of the parental cells PC9 and its derived highly BM subgroup PC9-BrM3, which was established by injecting PC9 into the left-ventricle of immunodeficient mice and isolating the metastatic cells from harvested brain metastases three times repeatedly in our previous work, for proteomics [20]. The RSD distribution of QC samples showed good repeatability of the proteomics data (Fig. 2a). Using ratio folds (BrM3/PC9) > 1.3 as the screening criteria to identify the differential proteins in the BM subgroup, 773 proteins were found upregulated and 587 proteins were found downregulated compared to the parental PC9 cells (Fig. 2b). Bioinformatics analysis suggested significant changes in proteins involved in the binding and catalytic ability in BM, including CTSF and FBLN1, which were finally identified (Fig. S1). Among these proteins, we focused on 10 proteins (CTSF, FBLN1, AKR1B10, CCL20, SAA1, CXCL1, CXCL3, AXL, AKR1C3, CPNE 3) based on the extent of upregulation and their close relationship with the occurrence and development of tumours reported in previous studies (Fig. 2c). ELISA was used to verify the above proteins in the serum of a small cohort of clinical samples [cohort 1: 20 NSCLC BM (LCBM), 20 advanced NSCLC without distant organ metastasis (ALC), 20 early-stage NSCLC (ELC), and 20 healthy groups (HG) patients]. There were no significant differences between the subgroups in cohort 1 with respect to age or sex (Table S1). Among them, compared with the controls, the levels of CTSF, FBLN1 and AKR1B10 were significantly upregulated in LCBM (Fig. 2d). These findings suggested that CTSF, FBLN1 and AKR1B10 are potential diagnostic markers for NSCLC BM.

### NSCLC patients with BM showed elevated serum levels of FBLN1 and CTSF

The clinical value of CTSF, FBLN1, and AKR1B10 in the diagnosis of NSCLC BM was assessed in cohort 2 comprising of 459 patients including 379 patients with NSCLC, 30 primary brain tumours (PBT), and 50 HG. The 379 NSCLC patients included 204 LCBM, 40 single organ liver metastasis (LM), 50 single organ bone metastasis (BoM), 40 ALC, 45 ELC. There were no significant differences between the subgroups in this cohort with respect to age or sex (Table S2).

Serum levels of CTSF and FBLN1 were significantly elevated in LCBM compared to controls (*P* < 0.001 for both) (Fig. 2E, F) while AKR1B10 was elevated in all groups with advanced NSCLC (LCBM, LM, BoM and ALC) irrespective of BM (Fig. 2G). On comparing among each control group, serum CTSF was found to be significantly elevated in ELC compared to HG (*P* = 0.009), while the level of FBLN1 and AKR1B10 was not significantly different between ELC and HG (*P* = 0.293, *P* = 0.05). Both CTSF and FBLN1 were significantly elevated in patients with advanced NSCLC (LCBM, LM, BoM, ALC) (*P* < 0.001 for both) compared to ELC.

On analyzing the clinicopathological correlates of serum CTSF, FBLN1 and AKR1B10 levels in patients with NSCLC BM, CTSF showed a significant association with sex; the mean CTSF level in male patients was significantly higher than that in female patients (*P* < 0.001, Table S3). On subgroup analysis of NSCLC BM patients disaggregated by sex, although the average CTSF level in female patients was lower than that in male patients, the level of CTSF in female patients was still significantly higher than that in the control groups (*P* < 0.001, Fig. S2). FBLN1 was related to D-dimer levels (*P* = 0.033), while AKR1B10 was related to the number of lung primary lesions (*P* = 0.004). Of note, in patients with BM, their CTSF, FBLN1 and AKR1B10 levels were not related to the presence or absence of distant metastasis to other organs, whether the patient had undergone surgery for primary lung lesions before BM and other clinicopathological characteristics. We further evaluated the role of serum CTSF and FBLN1 in NSCLC BM through multivariable logistic regression analysis in three serum cohorts. The results were significantly indicated that serum CTSF and FBLN1 were independent factors of NSCLC BM (Fig. S3). In summary, these findings indicate the potential role of CTSF and FBLN1 as liquid biopsy diagnostic markers for NSCLC BM.

### Increased expressions of FBLN1 and CTSF in metastatic tissues of NSCLC BM patients

**Fig. 3:**
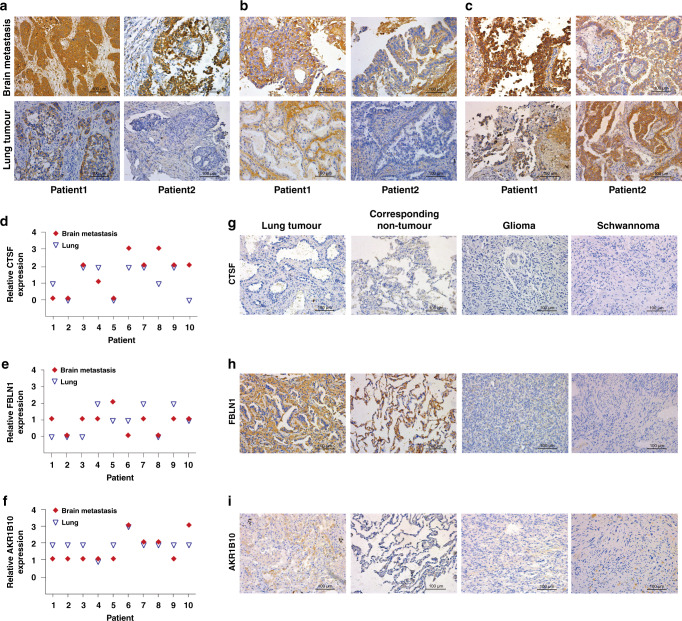
Tissue expressions of CTSF, FBLN1 and AKR1B10 in cohort 3. Cathepsin F (CTSF) (**a**), Fibulin-1 (FBLN1) (**b**) and Aldo-keto reductase family 1 member B10 (AKR1B10) (**c**) representative immunohistochemistry (IHC) staining of paired primary lung tumour and brain metastasis (BM) tissues from 2 patients in cohort 3. A representative area is selected. Relative expressions of CTSF (**d**), FBLN1 (**e**) and AKR1B10 (**f**) in primary lung tumour and BM tissues of 10 patients are shown. 0: negative, 1: weak positive, 2: positive, 3: strong positive. Representative IHC staining for CTSF (**g**), FBLN1 (**h**) and AKR1B10 (**i**) of patients from each control in cohort 3. Corresponding non-tumour is matched with non-small cell lung cancer patient without BM.

We examined the expressions of CTSF, FBLN1 and AKR1B10 in BM tissues of 47 patients with NSCLC BM using IHC (cohort 3); 10 of these patients underwent orthotopic tumour excision in the early stages of the disease and the corresponding primary tumour tissues were also obtained. Representative IHC staining of paired lung tumour and brain metastases from two patients is shown. CTSF and AKR1B10 staining were notably dominant in the cytoplasm of tumour cells (Fig. 3a, c) while FBLN1 staining was also observed in the interstitial cells in addition to the cytoplasm of tumour cells (Fig. 3b). Here the analysis of IHC results is based on the expression of the protein in tumour cells. Concordance of the expressions of CTSF, FBLN1 and AKR1B10 between paired lung tumours and brain metastases was shown in Fig. 3d–f. A relatively larger number of patients showed higher CTSF expression in brain metastases than that in the primary lung lesion as compared to patients who showed lower CTSF expression in brain metastases than that in the primary lung lesion. An opposite phenomenon was observed with respect to the expressions of FBLN1 and AKR1B10.

As controls, we examined tissue specimens of 15 NSCLC patients without BM and 13 corresponding non-tumour lung tissues, 9 glioma tissues and 5 Schwannoma tissues. The expression of CTSF in BM tissue was significantly higher than that in the controls (Table 1). CTSF was not commonly expressed in lung tissues and other brain tumours (Fig. 3g). The overall staining of FBLN1 in BM tissues was relatively weak. The ratio of strong positive and positive was low, and most of the tissues showed weak positive expression (Table 1). FBLN1 was also expressed in a certain proportion of lung tissues; however, it was not commonly expressed in other brain tumours (Fig. 3h). While, AKR1B10 was not specifically expressed in BM tissue, since the expression in brain metastases was not significantly different from NSCLC without BM and glioma tissues (Table 1 and Fig. 3i).

**Table 1:** Results of IHC showing tissue expressions of CTSF, FBLN1, and AKR1B10 in each clinical group in cohort 3.

Occur/total (%)									
	Brain metastasis	Glioma	*P*	Schwannoma	*P*	NSCLC without BM	*P*	Corresponding non-tumour	*P*
CTSF									
Strong positive	2/47 (4%)	0/9 (0%)	0.013*	0/5 (0%)	0.006**	0/15 (0%)	0.017*	0/13 (0%)	0.005**
Positive	21/47 (45%)	2/9 (22%)		0/5 (0%)		2/15 (13%)		0/13 (0%)	
Weak Positive	13/47 (28%)	0/9 (0%)		0/5 (0%)		3/15 (20%)		4/13 (31%)	
Negative	11/47 (23%)	7/9 (78%)		5/5 (100%)		10/15 (67%)		9/13 (69%)	
FBLN1									
Strong positive	0/47 (0%)	0/9 (0%)	0.001**	0/5 (0%)	0.003**	0/15 (0%)	0.031*	0/13 (0%)	0.224
Positive	5/47 (11%)	0/9 (0%)		0/5 (0%)		2/15 (13%)		0/13 (0%)	
Weak Positive	30/47 (64%)	1/9 (11%)		0/5 (0%)		4/15 (27%)		7/13 (54%)	
Negative	12/47 (26%)	8/9 (89%)		5/5 (100%)		9/15 (60%)		6/13 (46%)	
AKR1B10									
Strong positive	5/47 (11%)	0/9 (0%)	0.385	0/5 (0%)	0.016*	0/15 (0%)	0.323	0/13 (0%)	0.000***
Positive	19/47 (40%)	2/9 (22%)		0/5 (0%)		5/15 (33%)		0/13 (0%)	
Weak Positive	21/47 (45%)	6/9 (67%)		3/5 (60%)		10/15 (67%)		0/13 (0%)	
Negative	2/47 (4%)	1/9 (11%)		2/5 (40%)		0/15 (0%)		13/13 (100%)	

Results of immunohistochemistry (IHC) showing tissue expressions of Cathepsin F (CTSF), Fibulin-1 (FBLN1) and Aldo-keto reductase family 1 member B10 (AKR1B10) in each clinical group in cohort 3. Statistical analyses of IHC staining expression between non-small cell lung cancer (NSCLC) brain metastasis (BM) and each control are shown. Corresponding non-tumour is matched with 13 of 15 NSCLC without BM.

*P < 0.05.

**P < 0.01.

***P < 0.001.

We further analysed the relationship of the expressions of CTSF, FBLN1, and AKR1B10 with various clinicopathological characteristics of patients with NSCLC BM. CTSF expression in BM was associated with smoking (*P* = 0.018, Table S4) while FBLN1 expression was associated with the number of lung primary lesions (*P* = 0.020) and N stage (*P* = 0.021). These findings indicated that CTSF and FBLN1 are potential specific tumour histological markers for NSCLC BM and can help predict metastatic behaviour.

### A predictive diagnostic model for NSCLC BM was established by ROC curve analysis

**Fig. 4:**
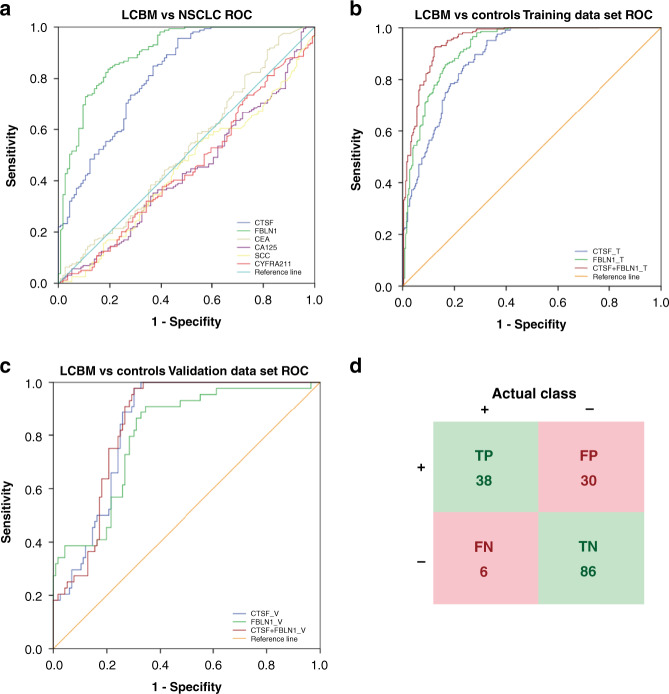
Receiver operating characteristic (ROC) curve analysis of Cathepsin F (CTSF) and Fibulin-1 (FBLN1). **a** ROC curve analysis of CTSF, FBLN1, carcinoembryonic antigen (CEA), carbohydrate antigen 125 (CA125), squamous cell carcinoma antigen (SCC) and cytokeratin fragment 21–1 (CYFRA21-1) for differentiating non-small cell lung cancer brain metastasis (LCBM) and non-small cell lung cancer (NSCLC) patients [single organ liver metastasis (LM), single organ bone metastasis (BoM), advanced NSCLC without distant organ metastasis (ALC) and early-stage NSCLC (ELC)] in cohort 2. ROC curve analysis of CTSF, FBLN1 and combination in patients with LCBM versus controls [LM, BoM, ALC, ELC, primary brain tumours (PBT) and healthy group (HG)] in the training dataset (**b**) and validation dataset (**c**). **d** The verification results of the validation dataset using the prediction model and cut-off value. TP true positive, FP false positive, FN false negative, TN true negative. Sensitivity = TP/(TP + FN) *100% = 86.36%; Specificity = TN/ (TN + FP) *100% = 74.14%.

ROC curve analysis was used to evaluate the performance of CTSF and FBLN1 as serum biomarkers for diagnosing NSCLC BM, compared with the classical serum biomarkers for lung cancer (CEA, CA125, SCC, CYFRA211). First, we analysed all NSCLC patients in cohort 2. As shown in Fig. 4a, the screening efficacy of CTSF (AUC = 0.813 cut-off value:76.25 sensitivity:95.6% specificity:53.5%) or FBLN1 (AUC = 0.899 cut-off value:111.04 sensitivity:83.6% specificity:80.7%) was better than that of CEA, CA125, SCC, and CYFRA211 as these classical markers could not distinguish BM from NSCLC (*P* > 0.05). These results indicated that CTSF and FBLN1 are potential diagnostic markers for BM. In order to establish a combined predictive diagnosis model of NSCLC BM based on serum levels of CTSF and FBLN1, we used patients in cohort 2 (LCBM and each control group) as the training dataset, and included another set of 160 patients (cohort 4: 44 LCBM and 116 controls) as validation dataset to verify the stability of the constructed predictive model. After combining CTSF with FBLN1 in a logistic regression model, the screening efficacy of the combination (AUC = 0.951 cut-off value:0.45 sensitivity: 92.6% specificity:87.5% Fig. 4b) was better than that of CTSF alone (AUC = 0.887 cut-off value:76.25 sensitivity:95.1% specificity:67.5%) and FBLN1 alone (AUC = 0.922 cut-off value:97.88 sensitivity:98.0% specificity: 71.4%). The predicted probability of NSCLC BM diagnosis from the stepwise logistic regression model was calculated as follows:

$${{{{{{{\mathrm{logit}}}}}}}}({{{{{{{\mathrm{P}}}}}}}}) = 0.027 \ast {{{{{{{\mathrm{CTSF}}}}}}}} + 0.048 \ast {{{{{{{\mathrm{FBLN}}}}}}}}1 - 8.530$$

The model was validated in the validation dataset. As shown in Fig. 4c, the AUC value of the combined model (AUC = 0.845 cut-off value:0.21 sensitivity:97.7% specificity:69.8%) was better than that of CTSF (AUC = 0.841 cut-off value:126.08 sensitivity:97.7% specificity:69.8%) or FBLN1 (AUC = 0.803 cut-off value:101.65 sensitivity:90.9% specificity:65.5%) alone. The serum concentration of patients in the validation dataset was factored in the prediction model. Using the cut-off value of 0.45, the sensitivity and specificity of the predictive diagnosis model in the validation dataset were 86.36% and 74.14%, respectively (Fig. 4d). Collectively, we established a predictive diagnosis model for NSCLC BM which showed that the combined analysis of serum CTSF and FBLN1 levels may facilitate the diagnosis of NSCLC BM. Thus, these markers may help fill a gap as the currently used clinical tumour markers cannot identify patients with BM.

### CTSF was identified as a follow-up biomarker to monitor the therapeutic response of BM patients

**Fig. 5:**
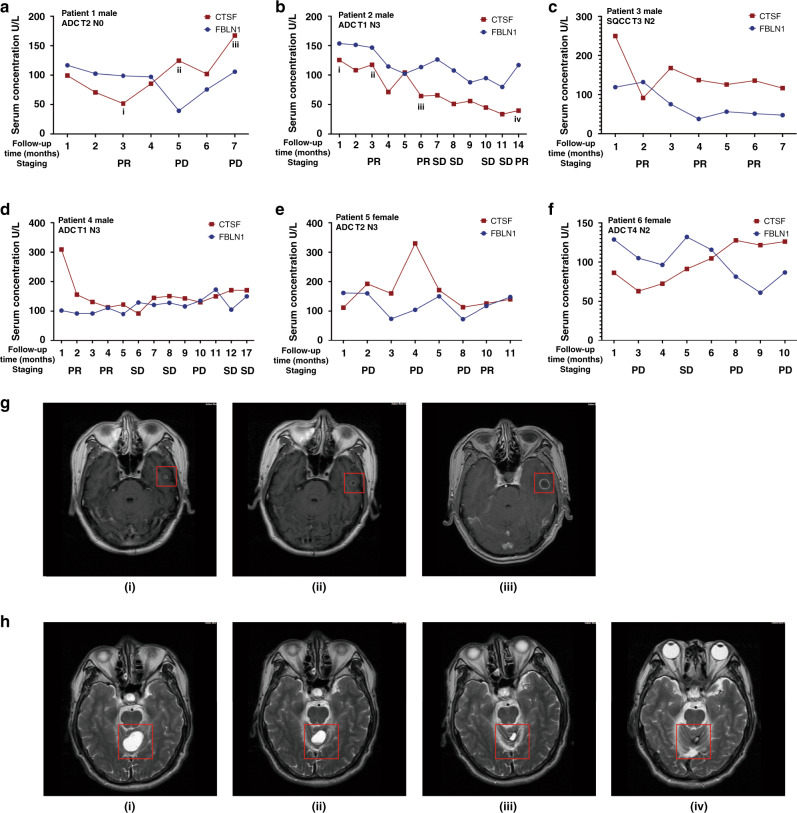
Serum CTSF predicts therapeutic response in NSCLC BM patients. Changes in serum concentrations of Cathepsin F (CTSF) and Fibulin-1 (FBLN1) in six non-small cell lung cancer (NSCLC) brain metastasis (BM) patients in cohort 2 during follow-up (**a**–**f**). Brain magnetic resonance images of patient 1 and patient 2 (**g, h**). The i/ii/iii/iv in A and B correspond to the i/ii/iii/iv in **g**, **h**. Response Evaluation Criteria in Solid Tumors1.1 was used to evaluate therapeutic efficacy. PR partial remission, SD stable disease, PD progression disease, ADC adenocarcinoma, SQCC squamous cell carcinoma, T tumour stage, N regional lymph Node stage.

To explore the potential association between the dynamic changes in serum CTSF and FBLN1 concentrations and treatment response in NSCLC BM patients, we regularly collected serum samples of 35 patients in cohort 2 during their clinical follow-up. In 25 of the 35 patients, the change in CTSF serum concentration was consistent with the change in treatment effect, and the predicted effective rate was 71.43%. Representative examples of the changes in serum concentrations during the disease course are shown in Fig. 5a–f. Figure 5g, h is the brain magnetic resonance images of patient 1 and patient 2, respectively. Results showed that the changes in serum levels of CTSF better reflected the patient’s response to treatment than FBLN1. For example, patient 1 was evaluated as having the progressive disease at 5–7 months; there was three-fold increase in the CTSF concentration during this period (Fig. 5a). The disease status of patient 2 was evaluated as partial remission at 3–6 months, and the CTSF concentration also decreased significantly (Fig. 5b). In these two patients, changes in serum FBLN1 concentration were similar to CTSF. Patient 4 was evaluated as partial remission at 1–4 months, and the CTSF concentration also decreased significantly; however, there was no significant change in the serum FBLN1 concentration (Fig. 5d). Patient 5 was evaluated as having the progressive disease at 1–4 months; there was three-fold increase in the CTSF concentration during this period, however, there was no significant change in the serum FBLN1 concentration (Fig. 5e). It was worth mentioning that the changes in serum levels of these markers preceded the changes in imaging findings by an average of ~1–3 months in 7 patients of cohort 2. For example, imaging findings of patient 1 (Fig. 5a) indicated disease progression after 5 months of treatment; however, the serum level increased at the 4th month. Patient 2 was evaluated as partial remission at the 14th month, however, the serum level decreased at the 11th month (Fig. 5b). Imaging findings of patient 4 indicated disease progression after 10 months of treatment; however, the serum level increased at the 7th month (Fig. 5d). Patient 6 was evaluated as having the progressive disease at the 8th months; however, the serum level increased at the 6th month (Fig. 5f). These data strongly suggest the benefit of frequent measurement of CTSF during therapy to predict the progression of the disease and make timely modifications in the treatment plan. These findings suggest that changes in serum CTSF level can be used to monitor the therapeutic response.

### CTSF predicts poor survival in NSCLC patients with BM

**Fig. 6:**
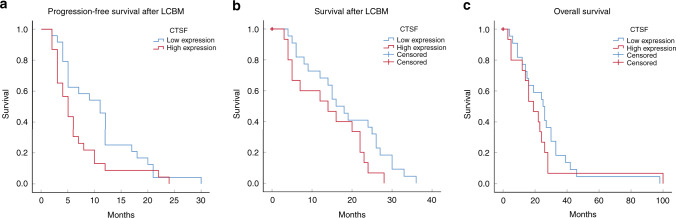
Prognostic potential of CTSF tissue expression in NSCLC BM patients. High expression includes strong positive and positive staining; Low expression includes weak positive and negative staining. Kaplan–Meier curve analysis of the relationship between Cathepsin F (CTSF) expression and progression-free survival after occurrence of non-small cell lung cancer (NSCLC) brain metastasis (BM) (**a**), survival after diagnosis of LCBM (**b**), overall survival from the time of diagnosis of NSCLC (**c**). LCBM = NSCLC BM patients.

In order to explore the prognostic relevance of CTSF in patients with NSCLC BM, we assessed the relationship between CTSF expression and survival of patients in cohort 3. All patients experienced disease progression and 83% (39 of 47) of patients had died at the time of completion of the analyses. The median PFS was 6.0 months while the median OS and survival after BM were 26.0 months and 22.0 months, respectively. According to the staining results, we categorised patients with strong positive and positive CTSF expression as the high expression group, while patients with weak positive and negative expression were categorised as the low expression group. PFS after the occurrence of BM was inferior in the CTSF high expression group compared with the CTSF low expression group [6.7 months 95% CI 4.362–9.116 vs.10.9 months 95% CI 8.041-13.792 *P* = 0.047 Fig. 6a]. Similarly, survival after the occurrence of BM tended to be inferior in the CTSF high expression group compared with the CTSF low expression group; however, owing to the small sample size, the difference was not statistically significant [13.9 months 95% CI 9.487–18.380 vs.18.4 months 95% CI 14.248–22.479 *P* = 0.071 Fig. 6b]. However, there was no significant between-group difference with respect to the OS from the time of diagnosis of NSCLC (26.5 months 95% CI 18.237–34.763 vs. 22.7 months 95% CI 11.136–34.331 *P* = 0.343 Fig. 6c).

Further, Cox proportional-hazards model was applied to assess the prognostic significance of CTSF in patients with NSCLC BM. The model satisfied the proportional-hazards assumption (Table S5). The risk of disease progression in the CTSF high expression BM patients was 2.052 times higher than that in CTSF low expression BM patients (HR = 2.052 95% CI 1.034–4.072 *P* = 0.04) after adjusting for age, sex, pathological type and smoke status. This indicated that CTSF was an independent prognostic factor for patients with NSCLC BM (Table S6).

## Discussion

Identification of non-invasive biomarkers for early diagnosis of NSCLC BM and dynamic monitoring of disease status is a key research imperative. There is a growing consensus that biomarker panels have higher specificity and sensitivity than single biomarkers and may be more effective in detecting cancer [21, 22]. Herein, we sought to discover the unique patterns of serum proteins in NSCLC patients with BM, and to identify biomarkers with sufficient sensitivity and specificity for use as a supplement or substitute for MRI in clinical practice. In the present study, CTSF and FBLN1 were identified as novel diagnostic biomarkers for NSCLC BM. Moreover, CTSF may be a sensitive biomarker for follow-up monitoring of therapeutic response, as well as a prognostic marker in patients with BM.

CTSF, also known as CATSF/CLN13, is a member of the cysteine cathepsins family. Cathepsins are key acid hydrolases within the lysosomes, and represent the main effectors of protein catabolism and autophagy [23]. The secreted cathepsins are recognised as effectors, which can modify the tumour microenvironment through the turnover and degradation of the extracellular matrix (ECM) [24], and by processing, activating or degrading various cytokines, growth factors, and chemokines [25, 26]. Cathepsins also shed inter-cellular adhesion molecules [27] and are involved in the regulation of angiogenesis [28], thereby promoting tumour cell metastasis. There is intertumoural heterogeneity and organ specificity of specific enzymes, and the functional relevance of each is highly dependent on the milieu [28, 29]. Expression of individual cathepsins can also help distinguish between metastatic pathways (venous or lymphatic) [30], and can identify metastatic involvement of specific sites. It remains unclear whether this differential tumour-metastatic activity of CTSF in different organs is attributed to tissue-specific substrates, or to alternative mechanisms of tissue-specific proteolytic regulation.

FBLN1, a widespread component of the ECM, can intervene in cell signal transduction events by binding to ECM proteins [31]. Studies have shown that FBLN1 can regulate cell morphology, adhesion, spread and promote cell movement, which is related to cancer growth, cell migration, and invasive growth [32–36]. The effect of FBLN1 as an ECM on cell migration and metastasis is complex [37]. On the one hand, the ECM is a storeroom of copious signal molecules, which can regulate various tumour-related pathological and physiological processes including tumour metastasis. Several extracellular proteases, such as matrix metalloproteinases, can process ECM into functional fragments and promote cell migration [38, 39]. On the other hand, the composition and structure of the ECM determine the resistance and adhesion encountered by the migrating cells, thereby increasing the colonisation of tumour cells in the blood vessel wall and target organs [40]. The effect of FBLN1 on cell migration and metastasis is believed to be specific to cell and organ types [37]. Therefore, FBLN1 may be involved in regulating the various stages of BM of tumour cells, such as detachment from the primary tumour, infiltration of blood vessels, and colonisation of the brain parenchyma. Further studies are required to verify the mechanisms.

This is the first study to show that the combination of serum CTSF and FBLN1 levels is a potential novel diagnostic biomarker panel for NSCLC BM patients. Firstly, we found that the serum concentrations of CTSF and FBLN1 in NSCLC BM patients were significantly higher than those in NSCLC patients without BM, patients with a primary brain tumour, and healthy individuals, which were not affected by the presence or absence of other organ metastasis or whether the patient had undergone lung lesion surgery before the BM. These findings suggest that the serum CTSF and FBLN1 are specific markers of BM in NSCLC patients. Although serum CTSF levels were significantly different between men and women, this did not affect their specific diagnostic ability. Of note, CTSF increased with the occurrence and progression of NSCLC. This indicated that different threshold expression levels of CTSF may facilitate the diagnosis of early-stage NSCLC and BM, and predict the risk of BM, similar to alpha fetal protein (AFP), a biomarker widely applied for the diagnosis of hepatocellular carcinoma (HCC). Additionally, previous studies have found downregulation of the level of FBLN1 in NSCLC [41]. Similarly, in our study, serum FBLN1 level in ELC was slightly lower than that in HG, while FBLN1 only significantly increased on the progression of NSCLC to an advanced stage.

Secondly, the expressions of CTSF and FBLN1 in NSCLC BM tissues were significantly higher than those in NSCLC without BM and other primary brain tumour tissues, although the expression of FBLN1 in tumour cells was relatively weak. However, FBLN1 was also widely expressed in lung tissues, while CTSF was not. In the lung tumours and its corresponding BM tissues, there was high consistency in the expressions of both CTSF and FBLN1. Higher expressions of CTSF and FBLN1 showed a strong correlation with clinicopathological factors such as smoking status, D-dimer levels, number of lung lesions and N stage. Thirdly, on ROC curve analysis, a combination of CTSF and FBLN1 was found to effectively discriminate NSCLC BM cases from NSCLC and primary brain tumours cases. Overall, the predictive model for the diagnosis of BM from NSCLC constructed in this study fills a critical gap as currently there are no specific tumour markers to identify patients with BM.

Several studies have found that cathepsins are a part of the dynamic response to anticancer therapy in the tumour microenvironment [42, 43], and can predict the response of breast and colorectal cancer to anticancer treatments [44–46]. On follow-up of patients with NSCLC BM in our study, we surprisingly found the changes in serum levels of CTSF preceded the changes in imaging findings by an average of approximately 1–3 months in 7 patients, which strongly suggest the benefit of frequent measurements of CTSF during therapy to detect disease progression. It is difficult to observe the sequence of serum and imaging changes in patients whose curative effect is evaluated as sustained remission and continuous progression. The sequence of changes in serum and imaging findings can only be judged in patients who exhibit changes in their condition. A well-designed and larger cohort study is warranted in the near future to confirm the predictability of CTSF on the progression trends in BM. Increased expression of cathepsin has been shown to be associated with poor prognosis in patients with ovarian, lung, breast, colorectal, head and neck cancers, and melanoma [47–49]. These findings are consistent with those of the present study wherein elevated CTSF expression was associated with poor PFS. Moreover, CTSF expression was observed as an independent prognostic factor. Assessment of the expression of CTSF in NSCLC patients at the time of diagnosis of BM may help predict the prognosis of patients. Although analysis of the resected tissues is invaluable in establishing the utility of expression of CTSF as a prognostic indicator, determining the circulating CTSF in serum is a convenient non-invasive method that can provide useful information about tumour malignancy.

Although serum levels of CTSF and FBLN1 seem to be promising biomarkers, some limitations of our study should be considered while interpreting the results. For example, there might be some inherent biases since clinical parameters are variable between institutions and individual clinicians. Therefore, a well-designed and large-scale multicenter follow-up cohort study is warranted to provide more robust evidence.

In summary, our study provides the first evidence that CTSF and FBLN1 are potential novel serum markers for the early diagnosis of patients with NSCLC BM. CTSF can also be used as a biomarker for assessing therapeutic efficacy and prognosis.

## Supplementary information

Supplementary Materials

Fig.s1

Fig.s2

Fig.s3

Table s1-s6

## Data Availability

The mass spectrometry proteomics data have been deposited to the ProteomeXchange Consortium (http://proteomecentral.proteomexchange.org/cgi/GetDataset) via the PRIDE partner repository with the dataset identifier PXD023721 (username: reviewer_pxd023721@ebi.ac.uk, password: a702THXE).
