# Supplementary material for: Cathepsin F and Fibulin-1 as novel diagnostic biomarkers for brain metastasis of non-small cell lung cancer
Source: Br J Cancer. 2022 Feb 25;126(12):1795–805. doi: 10.1038/s41416-022-01744-3 (PMC9174239; doi:10.1038/s41416-022-01744-3)
Supplement: Supplementary file 1 — Supplementary Materials [file 41416_2022_1744_MOESM1_ESM.docx]

**Supplementary Materials**

**Materials and Methods**

Study design

In the animal model construction and discovery phase, we conducted secretory proteomics in lung cancer cell line and its derived brain metastatic subpopulation which was enriched with BM characteristics by BM animal models as previous work ^[21]^, to screen potential biomarkers of BM. Ten candidates (CTSF, FBLN1, AKR1B10, CCL20, SAA1, CXCL1, CXCL3, AXL, AKR1C3 and CPNE3) identified by proteomics were validated in an initial serum cohort 1 (n=80) of 20 NSCLC BM (LCBM), 20 advanced NSCLC without distant organ metastasis (ALC), 20 early-stage NSCLC (ELC), and 20 healthy group (HG) patients. Three of these candidates were strongly suggested to possess potential diagnostic value for BM. In the experimental verification phase, further assessment was performed in two independent cohorts (cohorts 2 and 3) of patients with NSCLC BM and corresponding controls. And refer to the diagnostic sensitivity and specificity in cohort 1 to calculate the sample size included in the verification cohort. In cohort 2 [n=459, 204 LCBM, 40 single organ liver metastasis (LM), 50 single organ bone metastasis (BoM), 40 ALC, 45 ELC, 30 patients with primary brain tumors (PBT), and 50 HG] serum samples were detected, while in cohort 3 (n=76, 47 LCBM, 15 NSCLC without BM, 13 corresponding non-tumor lung tissue of 15 NSCLC without BM, 9 Glioma and 5 Schwannoma) tissue specimens were detected since patients in this cohort qualified the indications for surgery. In order to determine the diagnostic performance, a logistic regression model was adopted to develop a panel of the targeted biomarkers using training dataset (cohort 2). Subsequently, the performance of the logistic model was validated in a validation dataset (cohort 4; n=160, 44 LCBM, 22 LM, 25 BoM, 8 ALC, 22 ELC, 15 PBT, 24 HG). In the follow-up monitoring phase, representative patients in cohort 2 were followed up to monitor the therapeutic effect. Finally, in the prognostic evaluation phase, we evaluated the prognostic value of CTSF by calculating the progression-free survival (PFS) and overall survival (OS) of patients in cohort 3.

Animal studies

Animal models of lung cancer BM were established as previously described ^[21]^. In brief, the NSCLC cell line PC9 was engineered to stably express GFP- luciferase fusion protein through transfection of a triple modality plasmid vector. Twenty athymic female BALB-c-nu mice aged between 4 and 6 weeks were purchased from Beijing Vital River Laboratory Animal Technology Co., Ltd., China. Since our previous work presented that the BM efficiencies of parental PC9 and BM subpopulations were more than 20%, 20 mice were enough to guarantee the occurrence of BM events. The mice are kept in a Specific Pathogen Free animal room. After anesthetizing with ketamine (100 mg/kg body weight; Sigma, USA) and xylazine (10 mg/kg body weight; Sigma, USA), approximately 10^6^ transfected cells in 100 ml PBS were injected into the left ventricle of each mouse. Brain colonization was analyzed *in vivo* by bioluminescence imaging (BLI) which was conducted blindly and weekly. After retro-orbital injection of D-Luciferin (150 mg/kg body weight; Promega, USA), images were acquired with an IVIS Spectrum Xenogen machine (PerkinElmer, USA). The Living Image software (version 2.50) was used to analyze the bioluminescence images. BM-burden mice were sacrificed by spinal dislocation euthanasia after brain metastases appeared while BM-free mice were sacrificed at the ninth week after intracardiac injection. No randomization was used in animal studies.

Proteomics

Protein extraction

After 24 hours of serum-free culture, and when the cell density was > 80% of the culture dish, the supernatants of PC9 and BrM3 cell lines were collected by centrifugation. Solid impurities were removed by centrifugation at 12000 g at 4°C for 10 min. Then the supernatant was transferred to an ultrafiltration centrifuge tube (Millipore), and concentrated to 0.5 mL by centrifugation at 5000 g at 4°C. The protein was dissolved in buffer (8M urea), and the protein concentration was determined using the BCA kit.

Trypsin digestion

The same amount of protein was taken from each sample for enzymatic hydrolysis. An appropriate amount of standard protein was added and the volume adjusted to the same level using the lysis buffer. 20% TCA was slowly added to the final concentration followed by vortex to mix, and precipitation at 4°C for 2 hours. After centrifugation at 4500 g for 5 min, the supernatant was discarded, and the precipitate washed 2–3 times with pre-cooled acetone. After drying the pellet, TEAB was added with a final concentration of 200 mM. The pellet was ultrasonically dispersed followed by addition of trypsin at a ratio of 1:50 (protease: protein, m/m), and overnight hydrolysis. Dithiothreitol (DTT) was added to a final concentration of 5 mM, and reduced at 56°C for 30 min. Subsequently, iodoacetamide (IAA) was added to make the final concentration 11 mM, which was incubated for 15 min at room temperature in dark.

TMT labeling

After trypsinization, the peptides were desalted using a Strata X C18 SPE column (Phenomenex) and vacuum dried. The peptide was redissolved in 0.5 M TEAB and processed according to the manufacturer's TMT kit operating procedures. In short, one unit of TMT reagent was thawed and reconstituted in acetonitrile. The peptide mixture was then incubated for 2 hours at room temperature, combined, desalted, and dried by vacuum centrifugation.

HPLC fractionation

The sample was then separated by high pH reversed phase high-performance liquid chromatography (HPLC) using an Agilent 300Extend C18 column (5 μm particles, 4.6 mm ID, 250 mm length). In brief, the peptide was first separated into 60 fractions with a gradient of 8%–32% acetonitrile (pH 9.0) within 60 minutes. Then, the peptides were combined into 14 fractions and dried by vacuum centrifugation.

Liquid chromatography–mass spectrometry (LC-MS/MS) analysis

The trypsin peptide was dissolved in 0.1% formic acid and 2% acetonitrile (solvent A), and the sample was directly loaded on a self-made reversed-phase analytical column. The gradient included: increasing from 8% solvent B (0.1% formic acid in 90% acetonitrile) to 22% in 20 minutes, increasing from 22% to 35% in 7 minutes, and increasing to 80% in 4 minutes, followed by maintenance at 80% on the EASY-nLC 1000 UPLC system, and performed at a constant flow rate of 600 nL/min for the last 3 minutes. The peptide was subjected to an NSI source; the peptide was subjected to nanospray ionization and the applied electrospray voltage was 2.0 kV. Then tandem mass spectrometry (MS/MS) was performed in Q Exactive Plus (Thermo Scientific) connected online to UPLC. The m/z scan range was 400 to 1500 for full scan, and the complete peptide was detected in Orbitrap with resolution Is 70,000. The scan range of the secondary mass spectrum was set to a fixed starting point of 100 m/z, and the secondary scan resolution was set to 17,500. In the MS survey scan with dynamic exclusion of 30.0 s, for the first 20 precursor ions above the threshold ion count of 10,000, a data-dependent procedure was applied that alternated between one MS scan and the subsequent 20 MS/MS scans. In order to improve the utilization of the mass spectrometer, the automatic gain control (AGC) was set to 5E4, the signal threshold was set to 63000 ions/s, and the maximum injection time was set to 80 ms. The fixed first mass setting was 100 m/z.

Database search

The obtained MS/MS data was processed using the Maxquant search engine (v.1.5.2.8). The database used was Homo_sapiens_9606_SP_20191115 (20380 sequences), and an anti-database was added to calculate the false positive rate (FDR) caused by random matches. In order to eliminate the protein contamination in the identification results, a common contamination library was added to the database. Trypsin/P was designated as a lyase, allowing up to 2 missing cleavages. The minimum peptide length was set to 7 amino acid residues. The maximum number of peptide modifications was set to 5. For the main precursor ion of the first search, the mass error tolerance was set to 20 ppm, for the main precursor ion of the main search, the mass error tolerance was set to 5 ppm, and for the second fragment ion, the mass tolerance was set to 0.02 Da. The alkylation of cysteine was designated as a fixed modification, and the oxidation of methionine, acetylation and deamidation (NQ) of the N-terminus of the protein were designated as variable modifications. For the protein quantification method, TMT-6-plex was selected in Maxquant. For protein identification and PSM identification, FDR was adjusted to 1%.

Sample collection

Surgical specimens were obtained after surgical resection. At the time of the initial diagnosis, peripheral blood samples of all participants were collected for protein testing prior to the initiation of anti-tumor therapy. Serum samples were collected according to standard operating procedures. In short, peripheral blood samples (2 mL per subject) were collected in a serum separation tube, allowed to clot for 30 minutes, and then centrifuged at approximately 1,000 g/min for 15 minutes. Immediately after centrifugation, the serum was transferred to a clean polypropylene test tube and stored at -80°C.

Enzyme Linked Immunosorbent Assay (ELISA)

In brief, standards or samples were added to the wells of the antibody precoated microtiter plate in triplicate. After incubation, the biotinylated antibody and bound streptavidin-HRP were added to form an antibody-antigen-enzyme-antibody complex. After thorough washing, we added TMB substrate solution. After stopping the reaction with the stop solution, a color change was observed. After setting the blank well to zero, the optical density (OD) was measured at 450 nm using a microtiter plate reader. The OD value was proportional to the protein concentration with a standard curve.

**Figure legends**

**Fig. S1** GO function enrichment of differential proteins. GO function enrichment of up-regulated proteins (A) and down-regulated proteins (B).

**Fig. S2** Serum CTSF levels of different genders. Serum Cathepsin F (CTSF) concentration in male patient with non-small cell lung cancer brain metastasis (LCBM), female patient with LCBM and the corresponding control. Statistical analyses of serum concentrations between male patient with LCBM or female patient with LCBM and the corresponding control are shown. LCBM = non-small cell lung cancer (NSCLC) brain metastasis (BM); LM = single organ liver metastasis; BoM = single organ bone metastasis; ALC = advanced NSCLC without distant organ metastasis; ELC = early-stage NSCLC; PBT = primary brain tumors; HG = healthy group. The graphs show the summary of the results of 3 independent experiments.

**Fig. S3** Multivariable logistic regression analysis on the role of serum Cathepsin F (CTSF) and Fibulin-1 (FBLN1) in non-small cell lung cancer (NSCLC) brain metastasis (BM) through three serum cohorts. The analysis of cohort 1 (20 NSCLC BM vs 20 advanced NSCLC without BM) (A), cohort 2 (204 NSCLC BM vs 130 advanced NSCLC without BM) (B), cohort 4 (44 NSCLC BM vs 55 advanced NSCLC without BM) (C). The score of extent of extracranial metastatic disease ranged from 0-6 (liver, bone, lung metastases in addition to the primary, distant lymph nodes, adrenal gland, other sites). OR = odd ratio; CI = confidence intervals. ***P < 0.001; *P < 0.05.
