# Supplementary figures and images for: Cathepsin F and Fibulin-1 as novel diagnostic biomarkers for brain metastasis of non-small cell lung cancer
Source: Br J Cancer. 2022 Feb 25;126(12):1795–805. doi: 10.1038/s41416-022-01744-3 (PMC9174239; doi:10.1038/s41416-022-01744-3)

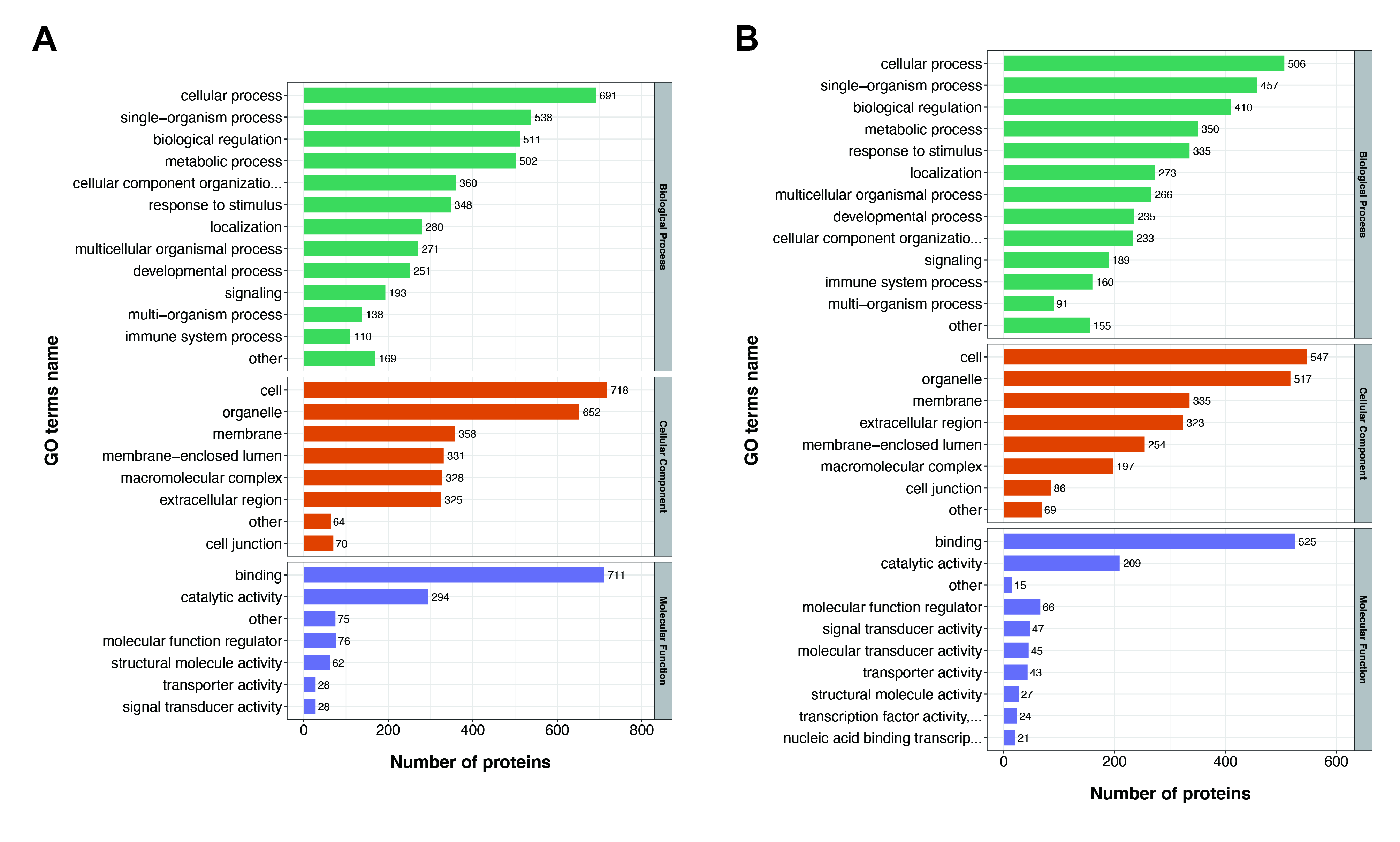

Supplement: Supplementary file 2 — Fig.s1 [file 41416_2022_1744_MOESM2_ESM.tif]

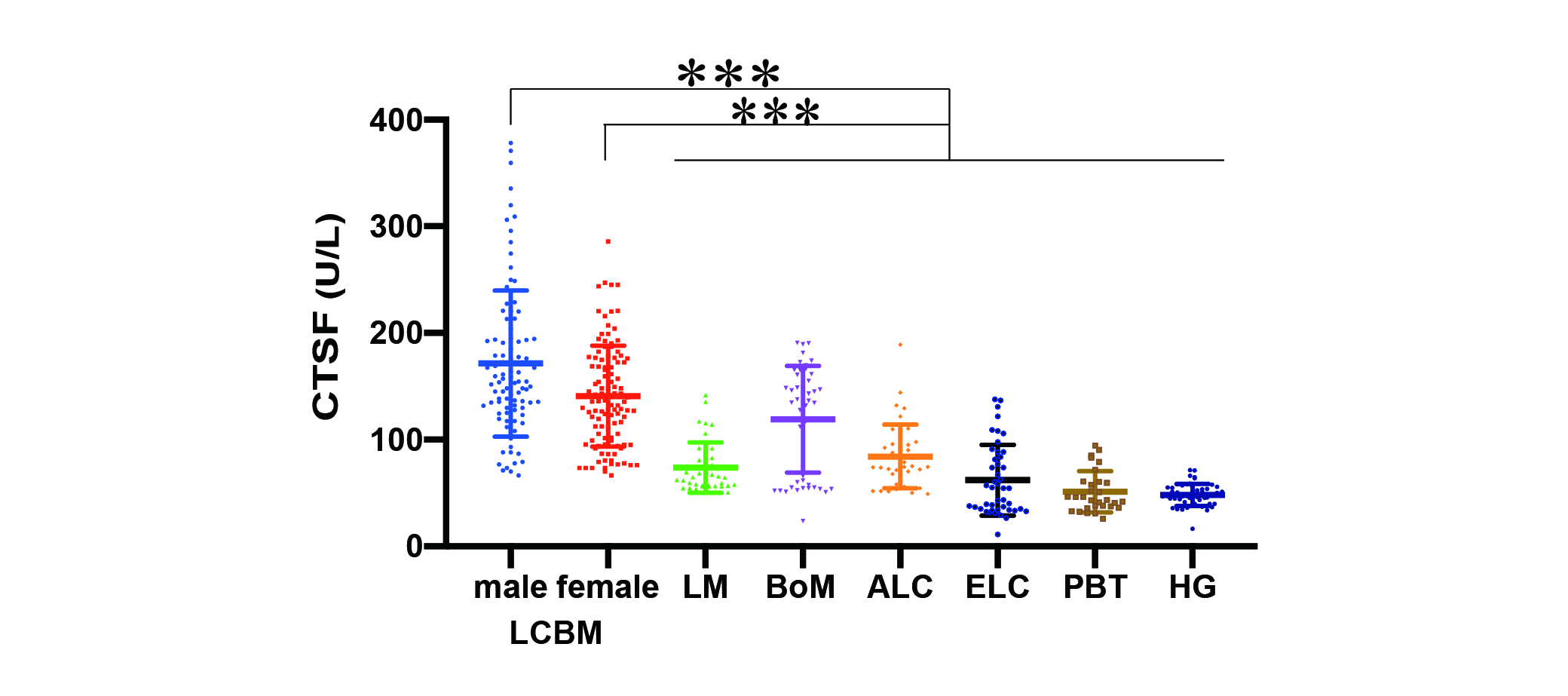

Supplement: Supplementary file 3 — Fig.s2 [file 41416_2022_1744_MOESM3_ESM.tif]

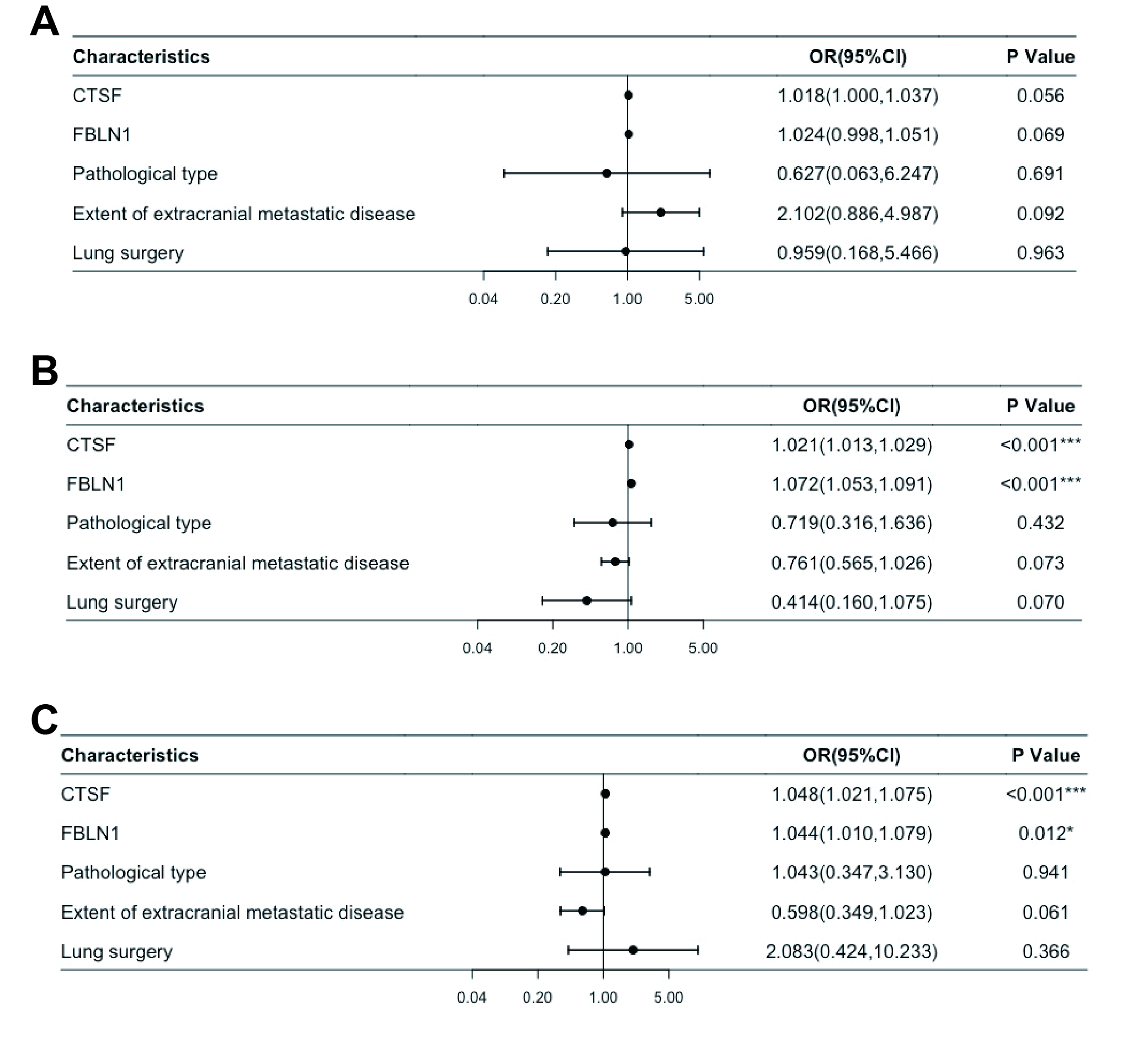

Supplement: Supplementary file 4 — Fig.s3 [file 41416_2022_1744_MOESM4_ESM.tif]
