# Supplementary material for: Cathepsin F and Fibulin-1 as novel diagnostic biomarkers for brain metastasis of non-small cell lung cancer
Source: Br J Cancer. 2022 Feb 25;126(12):1795–805. doi: 10.1038/s41416-022-01744-3 (PMC9174239; doi:10.1038/s41416-022-01744-3)
Supplement: Supplementary file 5 — Table s1-s6 [file 41416_2022_1744_MOESM5_ESM.docx]

Table S1. Baseline clinical characteristics of subjects in cohort 1.

| **Table S1.** |  |
| --- | --- |
| Characteristics | Case |
| Gender |  |
| Male | 42 |
| Female | 38 |
| Age (Year) |  |
| ≤60 | 27 |
| >60 | 53 |
| Pathological type |  |
| Adenocarcinoma | 55 |
| Squamous cell carcinoma | 5 |

Table S2. Baseline clinical characteristics of subjects in cohort 2

| **Table S2.** |  |  |  |
| --- | --- | --- | --- |
| Characteristics  stics | Case | Characteristics | Case |
| Lung cancer brain metastasis | 204 | Controls | 255 |
| Gender |  | Gender |  |
| Male | 95 | Male | 149 |
| Female | 109 | Female | 106 |
| Age (Year) |  | Age (Year) |  |
| ≤60 | 80 | ≤60 | 141 |
| >60 | 124 | >60 | 114 |
| Other organ metastasis |  | Type |  |
| Without | 127 | NSCLC | 175 |
| With | 77 | Glioma | 30 |
|  |  | Healthy group | 50 |
| Pathological type |  | Pathological type |  |
| Adenocarcinoma | 170 | Adenocarcinoma | 134 |
| Squamous cell carcinoma | 29 | Squamous cell carcinoma | 37 |
| Other types | 5 | Other types | 4 |
| Number of brain metastatic lesions |  | TNM stage |  |
| 1 | 94 | I / II | 45 |
| ≥2 | 110 | III / IV | 130 |
| Maximum diameter of BM (cm) |  | Organ metastasis |  |
| ≤2 | 140 | Liver | 40 |
| >2 | 64 | Bone | 50 |
| Meningeal metastasis |  | M stage |  |
| Without | 170 | M0 | 45 |
| With | 34 | M1 | 130 |
| T stage |  | T stage |  |
| T1 | 32 | T1 | 36 |
| T2 | 51 | T2 | 34 |
| T3 | 33 | T3 | 19 |
| T4 | 88 | T4 | 86 |
| N stage |  | N stage |  |
| N0 | 43 | N0 | 66 |
| N1 | 6 | N1 | 20 |
| N2 | 69 | N2 | 33 |
| N3 | 86 | N3 | 56 |

BM = brain metastasis. T = Tumor, N = regional lymph Node.

Table S3. Relationship of serum CTSF, FBLN1, and AKR1B10 levels with clinical characteristics of subjects in cohort 2.

| **Table S3.** |  |  |  |  |
| --- | --- | --- | --- | --- |
| Characteristics | Case | CTSF_*P* | FBLN1_*P* | AKR1B10_*P* |
| Gender |  |  |  |  |
| Male | 95 | ＜0.001*** | 0.142 | 0.893 |
| Female | 109 |  |  |  |
| Age (Year) |  |  |  |  |
| ≤60 | 80 | 0.078 | 0.782 | 0.500 |
| >60 | 124 |  |  |  |
| Pathological type |  |  |  |  |
| Adenocarcinoma | 170 | 0.073 | 0.959 | 0.533 |
| Squamous cell carcinoma | 29 |  |  |  |
| Other types | 5 |  |  |  |
| Smoke |  |  |  |  |
| Without | 141 | 0.062 | 0.129 | 0.568 |
| With | 63 |  |  |  |
| Other organ metastasis |  |  |  |  |
| Without | 127 | 0.252 | 0.725 | 0.152 |
| With | 77 |  |  |  |
| Number of brain metastatic lesions |  |  |  |  |
| 1 | 94 | 0.729 | 0.336 | 0.723 |
| ≥2 | 110 |  |  |  |
| Maximum diameter of BM (cm) |  |  |  |  |
| ≤2 | 140 | 0.545 | 0.223 | 0.363 |
| >2 | 64 |  |  |  |
| Meningeal metastasis |  |  |  |  |
| Without | 170 | 0.056 | 0.061 | 0.897 |
| With | 34 |  |  |  |
| Number of lung primary lesions |  |  |  |  |
| 1 | 116 | 0.472 | 0.328 | 0.004** |
| ≥2 | 88 |  |  |  |
| Maximum diameter of LP (cm) |  |  |  |  |
| ≤2 | 77 | 0.525 | 0.439 | 0.073 |
| >2 | 127 |  |  |  |
| Pleural effusion |  |  |  |  |
| Without | 128 | 0.542 | 0.378 | 0.100 |
| With | 76 |  |  |  |
| Lung surgery |  |  |  |  |
| Without | 146 | 0.978 | 0.612 | 0.613 |
| With | 58 |  |  |  |
| TNM stage |  |  |  |  |
| T1 | 32 | 0.149 | 0.664 | 0.184 |
| T2 | 51 |  |  |  |
| T3 | 33 |  |  |  |
| T4 | 88 |  |  |  |
| N stage |  |  |  |  |
| N0 | 43 | 0.785 | 0.346 | 0.526 |
| N1 | 6 |  |  |  |
| N2 | 69 |  |  |  |
| N3 | 86 |  |  |  |
| D-dimer |  |  |  |  |
| 0-1 ug/mL | 137 | 0.524 | 0.033* | 0.366 |
| >1 ug/mL | 67 |  |  |  |
| Fibrinogen |  |  |  |  |
| ≤4 g/L | 84 | 0.323 | 0.811 | 0.331 |
| >4 g/L | 120 |  |  |  |
| EGFR |  |  |  |  |
| Wild type | 21 | 0.204 | 0.091 | 0.508 |
| Mutant | 76 |  |  |  |

There are some missing values for EGFR results. Cathepsin F (CTSF), Fibulin-1 (FBLN1), Aldo-keto reductase family 1 member B10 (AKR1B10). BM = brain metastasis. T = Tumor, N = regional lymph Node. ****P* < 0.001; ** *P* < 0.01; * *P* < 0.05.

Table S4. Relationship of tissue expressions of CTSF, FBLN1, and AKR1B10 with clinical characteristics of subjects in cohort 3.

| **Table S4.** | | | | |
| --- | --- | --- | --- | --- |
| Characteristics | Case | CTSF_*P* | FBLN1_*P* | AKR1B10_*P* |
| Gender |  |  |  |  |
| Male | 24 | 0.097 | 0.112 | 0.516 |
| Female | 23 |  |  |  |
| Age (Year) |  |  |  |  |
| ≤60 | 21 | 0.214 | 0.492 | 0.384 |
| >60 | 26 |  |  |  |
| Pathological type |  |  |  |  |
| Adenocarcinoma | 43 | 0.140 | 0.058 | 0.725 |
| Squamous cell carcinoma | 4 |  |  |  |
| Other types | 0 |  |  |  |
| Smoke |  |  |  |  |
| Without | 30 | 0.018* | 0.164 | 0.752 |
| With | 17 |  |  |  |
| Number of brain metastatic lesions |  |  |  |  |
| 1 | 31 | 0.735 | 0.509 | 0.093 |
| ≥2 | 16 |  |  |  |
| Maximum diameter of BM (cm) |  |  |  |  |
| ≤2 | 16 | 0.759 | 0.685 | 0.089 |
| >2 | 31 |  |  |  |
| Meningeal metastasis |  |  |  |  |
| Without | 45 | 0.726 | 0.553 | 0.380 |
| With | 2 |  |  |  |
| Number of lung primary lesions |  |  |  |  |
| 1 | 27 | 0.126 | 0.020* | 0.664 |
| ≥2 | 20 |  |  |  |
| Maximum diameter of LP (cm) |  |  |  |  |
| ≤2 | 19 | 0.266 | 0.613 | 0.153 |
| >2 | 28 |  |  |  |
| Lung surgery |  |  |  |  |
| Without | 29 | 0.097 | 0.952 | 0.426 |
| With | 18 |  |  |  |
| D-dimer |  |  |  |  |
| 0-1 ug/mL | 27 | 0.202 | 0.349 | 0.436 |
| >1 ug/mL | 8 |  |  |  |
| T stage |  |  |  |  |
| T1 | 9 | 0.383 | 0.554 | 0.148 |
| T2 | 18 |  |  |  |
| T3 | 6 |  |  |  |
| T4 | 14 |  |  |  |
| N stage |  |  |  |  |
| N0 | 20 | 0.464 | 0.021* | 0.357 |
| N1 | 4 |  |  |  |
| N2 | 22 |  |  |  |
| N3 | 1 |  |  |  |

There are some missing values for D-dimer results. Cathepsin F (CTSF), Fibulin-1 (FBLN1), Aldo-keto reductase family 1 member B10 (AKR1B10). BM = brain metastasis. T = Tumor, N = regional lymph Node. * *P* < 0.05.

Table S5. Time-Dependent Cox Regression Model to assess the proportional hazards assumption.

| **Table S5.** |  |  |  |  |  |  |  |
| --- | --- | --- | --- | --- | --- | --- | --- |
|  | B | SE | Wald | *P* | Exp(B) | 95% CI | |
| T_COV_ | 0.251 | 0.177 | 2.009 | 0.156 | 1.286 | 0.908 | 1.820 |
| Gender | -0.386 | 0.347 | 1.235 | 0.266 | 0.680 | 0.344 | 1.342 |
| Age | -0.090 | 0.326 | 0.077 | 0.782 | 0.914 | 0.482 | 1.731 |
| Pathological type | -0.167 | 0.664 | 0.063 | 0.802 | 0.846 | 0.230 | 3.111 |
| Smoke | 0.002 | 0.002 | 0.829 | 0.362 | 1.002 | 0.998 | 1.006 |

T_COV_ is the interaction between Cathepsin F expression and time.

Table S6. Cox proportional-hazards model to analyze the prognostic significance of CTSF in NSCLC with BM.

| **Table S6.** |  |  |  |  |
| --- | --- | --- | --- | --- |
|  | *P*-value | HR | 95% CI | |
| CTSF | 0.040* | 2.052 | 1.034 | 4.072 |
| Gender | 0.631 | 1.235 | 0.523 | 2.917 |
| Age | 0.959 | 0.983 | 0.520 | 1.861 |
| Pathological type | 0.989 | 1.008 | 0.316 | 3.218 |
| Smoke | 0.368 | 1.481 | 0.630 | 3.484 |

Cathepsin F (CTSF), non-small cell lung cancer (NSCLC), brain metastasis (BM). **P* < 0.05.
